# Effect of Conization Prior to Radical Hysterectomy on Overall and Progression-Free Survival in Early-Stage Cervical Cancer: A Propensity Score-Matched Analysis

**DOI:** 10.3390/cancers17203360

**Published:** 2025-10-18

**Authors:** Nutthanun Rachadech, Sunisa Phookiaw, Kittipat Charoenkwan

**Affiliations:** Division of Gynecologic Oncology, Department of Obstetrics and Gynecology, Faculty of Medicine, Chiang Mai University, Chiang Mai 50200, Thailand; tjrachadech@gmail.com (N.R.); sunisa.phookiaw@gmail.com (S.P.)

**Keywords:** cervical cancer, conization, radical hysterectomy, propensity score matching, overall survival, progression-free survival

## Abstract

**Objective**: To evaluate the association of preoperative conization prior to radical hysterectomy (RH) with overall survival (OS) and progression-free survival (PFS) in patients with early-stage cervical cancer. **Methods**: We conducted a retrospective cohort study involving patients with stage IB1 cervical cancer treated by RH at Maharaj Nakorn Chiang Mai Hospital, Faculty of Medicine, Chiang Mai University, Thailand, between January 2003 and December 2019. Propensity-score matching (1:1 ratio, caliper of 0.2 standard deviations) was used to balance covariates between patients who underwent conization (*n* = 314) and those who did not (*n* = 528). Survival outcomes were analyzed using Kaplan–Meier curves and the log-rank test. Subgroup analysis was also conducted on patients without high-risk histopathological factors, including pelvic node metastasis, parametrial invasion, and positive vaginal margins. **Results**: A total of 274 matched pairs were analyzed. Conization was significantly associated with improved OS (log-rank *p* = 0.03). For PFS, the benefit associated with conization was not demonstrated (log-rank *p* = 0.18). Similarly, subgroup analysis excluding patients with high-risk histopathological factors showed a significant association between conization and improved OS (log-rank *p* = 0.04) but not with PFS (log-rank *p* = 0.29). **Conclusions**: Conization prior to RH is significantly associated with improved OS in patients with early-stage cervical cancer, though its association with PFS remains inconclusive. These findings support the integration of conization into personalized surgical treatment strategies, with further prospective validation recommended.

## Simple Summary

Cervical cancer is a common cancer among women globally. For patients with early-stage disease, surgery to remove the uterus along with closely surrounding tissues (radical hysterectomy) is often the main treatment. An outpatient procedure called conization, which removes a portion of the cervix, is sometimes performed before this major surgery to confirm the diagnosis and assess the extent of cancer. Recent evidence suggests that conization performed before radical hysterectomy may also improve treatment outcome, but the benefits have not been fully understood and vary among studies. We reviewed data from patients treated at our hospital and compared those who had conization before radical hysterectomy with those who did not. We found that patients who had conization lived longer overall. These findings suggest that conization may provide an important protective effect and could help guide more personalized surgical planning for women with early-stage cervical cancer.

## 1. Introduction

Cervical cancer remains a significant global health issue, particularly in developing countries where access to effective screening and comprehensive treatment is limited. It ranks as the fourth most diagnosed cancer and is a leading cause of cancer-related mortality among women worldwide [1]. Surgical treatment, including radical hysterectomy (RH), remains the primary therapeutic option for patients diagnosed with early-stage cervical cancer. However, the optimal surgical approach, including the use of preoperative procedures such as cervical conization, continues to evolve [2].

Cervical conization, traditionally performed as a diagnostic and therapeutic tool for microinvasive disease or as part of fertility-sparing approaches, has recently drawn attention for its potential to improve surgical outcomes in patients with early-stage cervical cancer [2]. Prior observational studies suggest that preoperative conization may reduce tumor size, limit cancer dissemination during RH, and ultimately improve survival outcomes. Specifically, recent evidence suggests improved overall survival and decreased recurrence rates in patients undergoing conization prior to RH. Despite promising observational data, the survival benefit of preoperative conization remains incompletely understood and varies among studies. Furthermore, the specific subgroup of patients most likely to benefit from preoperative conization has not been clearly identified [3,4].

This study aimed to clarify the association between preoperative conization and survival outcomes in patients with early-stage cervical cancer undergoing RH. Using a rigorous propensity-score matching methodology, we examined both overall survival and progression-free survival among patients receiving conization compared to those who did not. Additionally, we explored outcomes specifically within a subgroup of patients without high-risk histopathological features.

## 2. Materials and Methods

### 2.1. Study Design and Patient Population

We conducted a retrospective cohort study of women with early-stage cervical carcinoma treated at Maharaj Nakorn Chiang Mai Hospital, Faculty of Medicine, Chiang Mai University, between January 2003 and December 2019. Eligible patients include: (1) International Federation of Gynecology and Obstetrics (FIGO) stage IB1 (2009) patients; (2) histological confirmation of squamous cell carcinoma, adenocarcinoma, or adenosquamous carcinoma patients; and patients that (3) underwent Querleu–Morrow Type B and C RH as primary treatment at our institution. Type B RH involves resection of the paracervical tissue at the level of the ureteral tunnel with partial resections of the rectouterine-rectovaginal and vesicouterine ligaments and vaginal cuff (~10 mm) without paravaginal tissue excision. Type C RH entails transection of the paracervical tissue at its junction with the internal iliac vessels, with complete resection of both rectouterine-rectovaginal and vesicouterine ligaments, and tailored vaginal cuff excision based on tumor extent [5,6,7]. Exclusion criteria were (1) received any neoadjuvant treatment, either chemotherapy or radiation therapy, before RH; (2) cervical cancer with pregnancy; (3) had incomplete pathological information; and (4) had not had disease relapse but were lost to follow-up within three months after RH. The Research Ethics Committee of the Faculty of Medicine, Chiang Mai University, approved this study (approval number 464/2024, dated 3 December 2024). The study hypothesized that preoperative conization could be associated with improved disease-free and overall survival in early-stage cervical cancer patients undergoing RH.

### 2.2. Exposure and Outcomes

The exposure of interest was the performance of conization before RH. At our institution, preoperative conization was solely carried out for diagnostic reasons, specifically to acquire definitive staging information to distinguish microinvasive disease from more advanced conditions. Loop electrosurgical excision procedure (LEEP) was the preferred method for cervical conization, and cold-knife conization (CKC) was acceptable. Board-certified gynecologic oncologists performed all RH procedures. In patients with conization, the RH was generally performed 6–8 weeks following conization. The primary outcomes were overall survival (OS), defined as the time from RH to death from any cause, censored at the last follow-up, and progression-free survival (PFS), defined as the time from surgery to first clinical or radiological recurrence as defined by the Response Evaluation Criteria in Solid Tumors (RECIST) [8,9], censored at the last follow-up assessment.

At our center, adjuvant radiotherapy was administered according to the presence of intermediate-risk factors, as defined by the Sedlis criteria, which combine three factors: LVSI, depth of stromal invasion, and tumor size [10]. Concurrent chemoradiation therapy (CCRT), consisting of pelvic external beam radiotherapy of 50.4 Gy in 28 fractions with weekly intravenous cisplatin 40 mg/m^2^ for 4–6 cycles, was given to patients with high-risk histopathological factors, including pelvic lymph node metastasis, parametrial invasion, and involved vaginal surgical margin [11]. After completing treatment, patients were regularly evaluated every three months for the first year, every four months during the second year, every six months during the third and fifth years, and annually thereafter, with clinical examinations and imaging studies as needed to monitor for any signs of recurrence.

We compared survival outcomes between patients who underwent conization and those who did not, adjusting for potential confounders (covariates).

### 2.3. Covariates

We initially selected covariates based on clinical relevance and literature review. These included clinical characteristics, such as age, parity, menopausal status, and adjuvant treatment (none vs. radiotherapy and/or chemotherapy), as well as pathological characteristics, including tumor size (calculated by summing the maximum pathological tumor diameter on the conization specimen and the RH specimen), histological subtype, histological grade, depth of cervical stromal invasion, lymphovascular space invasion (LVSI), vaginal metastasis, uterine corpus involvement, adnexal metastasis, pelvic lymph node metastases, parametrial involvement, and positive vaginal margin. All categorical variables were coded as factors with binary or multinomial levels as appropriate.

Before estimating the propensity score, we compared baseline characteristics between the conization and non-conization groups in the full cohort. Hypothesis testing for the comparison of covariates between the two groups was performed by using Student’s *t*-test for continuous variables and chi-squared or Fisher’s exact test for categorical variables. For each covariate, we computed standardized mean differences (SMD), with |SMD| < 0.10 considered an acceptable balance. Variables with |SMD| ≥ 0.10 were deemed imbalanced and included in the propensity-score model to control for confounding.

### 2.4. Handling of Missing Data

Variables with missingness exceeding 20% (e.g., tumor grade, LVSI, depth of invasion) with significant collinearity were excluded from the propensity-score model to maximize sample retention and minimize model variance. The remaining covariates each had fewer than 5% missing values; therefore, we performed a complete-case analysis for propensity-score estimation, excluding observations with any missing value in the selected covariates.

### 2.5. Propensity-Score Estimation and Matching

We estimated each patient’s propensity to undergo conization using a logistic regression model that incorporated the remaining covariates, including age, adjuvant treatment, tumor size, histological subtype, vaginal metastasis, uterine corpus involvement, adnexal metastasis, pelvic lymph node metastases, parametrial involvement, and positive vaginal margin. We subsequently matched the conization and non-conization patients 1:1 without replacement on the logit-transformed propensity score, using nearest-neighbor matching within a caliper of 0.2 × SD of the logit propensity score to ensure balanced covariates between groups. This method enhances the validity of our findings by mitigating selection bias and allowing for a more accurate comparison of survival outcomes.

We assessed post-match balance using SMD, variance ratios, and empirical cumulative density function (eCDF) distances, aiming for |SMD| < 0.10. Balance diagnostics and the associated Love plot were generated.

### 2.6. Survival Analysis

All survival analyses were conducted in the matched cohort, which retained an equal number of patients in both the treated (conization) and control (non-conization) groups. Kaplan–Meier curves estimating OS and PFS were generated. The log-rank test was employed to compare survival distributions between the groups, with a significance level set at *p* < 0.05. Subgroup analysis on the association between conization and OS and PFS of propensity-score-matched patients without high-risk histopathological factors, including pelvic node metastasis, parametrial invasion, and positive vaginal margin, was also performed. This comprehensive approach allows for a clearer understanding of the association between conization and survival outcomes in early-stage cervical cancer patients. All analyses were performed in R (version 4.5) [12]. Statistical significance was set at a two-sided *p*-value of less than 0.05.

## 3. Results

### 3.1. Patient Population and Baseline Characteristics

A total of 842 patients with FIGO 2009 stage IB1 cervical cancer met the inclusion criteria: 314 (37.3%) underwent preoperative conization, and 528 (62.7%) did not. Prior to matching, the conization group exhibited a smaller median tumor size (1.6 vs. 2.5 cm, *p* < 0.001), a greater proportion of squamous cell carcinoma (73.6% vs. 64.4%, *p* = 0.016), a lessor proportion of grade 3 tumor (7.7% vs. 18.6%, *p* = 0.001), a lower percentage of cancer metastasis to the vagina (7.0% vs. 16.9%, *p* < 0.001), parametrial invasion (11.2% vs. 19.7%, *p* = 0.002), and pelvic node metastasis (11.5% vs. 18.4%, *p* = 0.01), and were less likely to receive adjuvant therapy (31.2% vs. 50.0%, *p* < 0.001).

### 3.2. Conization Margin and Residual Tumor

Among 314 patients who underwent conization, the median cone depth was 0.50 cm (interquartile range [IQR] 0.28 cm) and the median cone width was 0.85 cm (IQR 0.50 cm), as determined by pathological examination. Negative margins on the conization specimen were achieved in 32/314 (10.2%), and 85/314 (27.1%) had no residual tumor at hysterectomy. The median time interval from conization to RH was 43 days.

Patients with positive cone margins exhibited a larger median diameter of cancerous lesions in conization specimens (0.85 cm vs. 0.70 cm, *p* = 0.005). There were no statistically significant differences in other histopathological features between patients with positive and negative cone margins, including histological type, histological grade, LVSI, depth of stromal invasion, pelvic node metastasis, parametrial invasion, and involved vaginal margin. Patients with positive cone margins had a higher percentage of residual cancer in RH specimens (75.9% vs. 43.8%, *p* < 0.001) and were more likely to require postoperative adjuvant treatment (32.7% vs. 12.5%, *p* = 0.03). However, there were no differences in OS (*p* = 0.22) and PFS (*p* = 0.17) between patients with positive and negative cone margins.

Patients with and without residual cancerous lesions in RH specimens had comparable lesion sizes on the conization specimens (0.84 cm vs. 0.80 cm, *p* = 0.86) and intervals from conization to RH (41.0 days vs. 46.5 days, *p* = 0.20). Those with residual cancer in RH specimens had a significantly higher incidence of pelvic node metastasis (14.4% vs. 3.6%, *p* = 0.01) and parametrial invasion (15.4% vs. 0.0%, *p* < 0.001). These patients were more likely to require postoperative adjuvant treatment (39.7% vs. 8.2%, *p* < 0.001). Notably, the OS and PFS were significantly poorer in the group with residual cancerous lesions compared to those without (*p* = 0.01, for both).

### 3.3. Survival Outcomes Comparison Between Patients With and Without Conization

**Figure 1:**
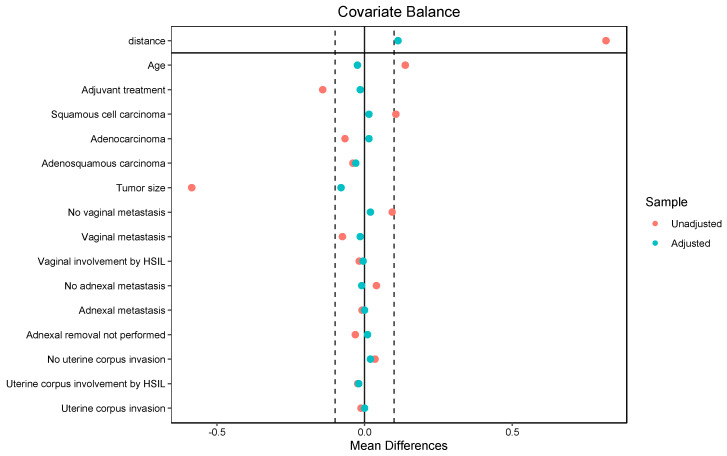
The Love plot illustrates the standardized mean differences (SMDs) of covariates between treatment groups before (red) and after (green) propensity score matching. The solid vertical line at zero represents perfect covariate balance, indicating no difference in means between groups. The dashed vertical lines at ±0.1 denote the conventional thresholds for acceptable imbalance. Covariates with SMDs within these limits are considered well balanced, demonstrating that matching effectively minimized baseline differences between groups.

After 1:1 propensity-score matching, which resulted in a matched cohort of 548 patients, 274 patients with conization were matched to 274 controls. Post-match, key covariates were well balanced (Table 1 and Figure 1).

**Figure 2:**
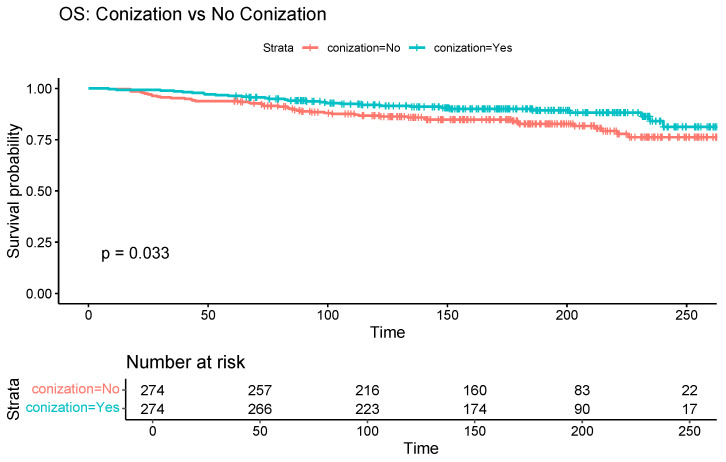
Kaplan–Meier curve comparing overall survival between patients with and without preoperative conization.

**Figure 3:**
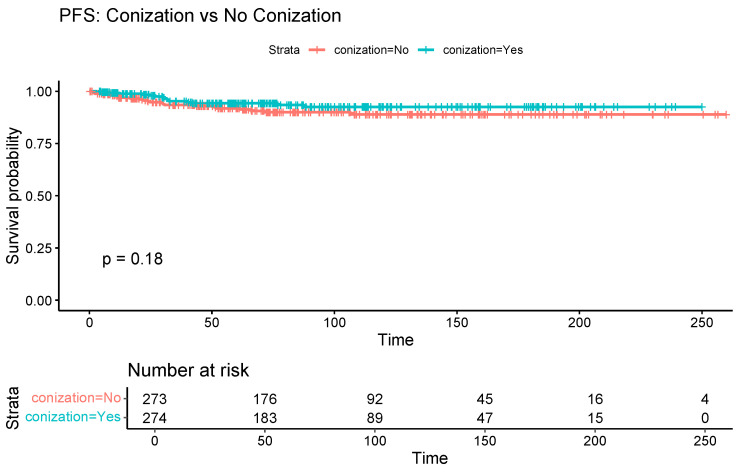
Kaplan–Meier curve comparing progression-free survival between patients with and without preoperative conization.

Table 2 compares oncological outcomes between the two groups. The median follow-up duration was 73.6 months in the conization group and 73.2 months in the control group. Kaplan–Meier curves showed a significant association between conization and improved OS (log-rank *p* = 0.03) (Figure 2). However, the association between conization and PFS was not statistically significant (log-rank *p* = 0.18) (Figure 3).

### 3.4. Subgroup Analysis Excluding Patients with High-Risk Histopathological Factors

Among 584 patients without high-risk histopathological factors, which include pelvic node metastasis, parametrial invasion, and positive vaginal margin, 404 patients were propensity-score matched with 202 patients per group after matching. The post-match covariate balance was excellent. Table 3 compares oncological outcomes between the two groups.

**Figure 4:**
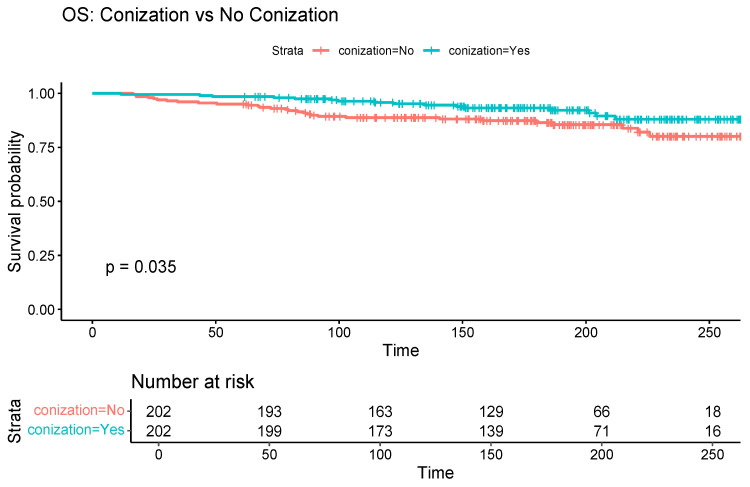
Kaplan–Meier curve illustrating the overall survival differences between patients with and without preoperative conization, among those without high-risk histopathological factors.

**Figure 5:**
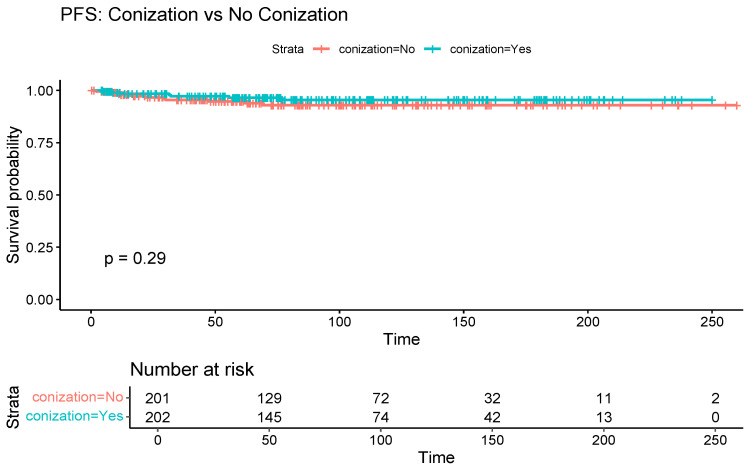
Kaplan–Meier curve illustrating the progression-free survival differences between patients with and without preoperative conization, among those without high-risk histopathological factors.

Similarly, the significant benefit of conization on OS (log-rank *p* = 0.04) but not on PFS (log-rank *p* = 0.29) was demonstrated (Figure 4 and Figure 5).

## 4. Discussion

In this propensity-score matched analysis of 274 patient pairs, preoperative conization was significantly associated with improved OS, although no significant difference in PFS was observed. Subgroup analysis excluding patients with high-risk histopathological features also demonstrated a significant association between conization and OS, while PFS remained comparable between groups.

Minimally invasive RH was considered a promising approach in managing patients with early-stage cervical cancer, offering benefits such as reduced recovery time and less postoperative pain, according to the data from retrospective studies [13,14]. However, the Laparoscopic Approach to Cervical Cancer (LACC) randomized controlled trial published in 2018 reported a significantly lower disease-free survival and OS in FIGO 2009 stage IA-IB1 cervical cancer patients who received minimally invasive RH compared to those who underwent open surgery [15]. This raised concerns about the safety of minimally invasive techniques in this population, necessitating further investigation into the optimal surgical approach for early-stage cervical cancer. It has been hypothesized that uterine manipulator use and CO2 pneumoperitoneum, leading to increased risk of cancer dissemination during intracorporeal colpotomy, could be causative factors of the observed inferior outcomes with minimally invasive surgery [16,17,18,19]. Still, a recent meta-analysis showed that the use of MIS without a uterine manipulator also resulted in an inferior recurrence-free survival when compared with open RH in the treatment of women with early-stage cervical cancer [20].

Data from recent studies have suggested that preoperative conization is associated with improved survival outcomes in early-stage cervical cancer patients [21,22,23,24,25,26,27]. In theory, preoperative conization may decrease tumor size, lowering the risk of the tumor coming into contact with the abdominal cavity and vaginal vault, hence potentially reducing disease recurrence in the vaginal area and surrounding organs [28]. In a recent meta-analysis including 12 retrospective studies focusing on the efficacy of preoperative conization in patients with early-stage cervical cancer [3], conization was associated with a significant improvement in disease-free survival when compared to patients who did not undergo conization. The hazard ratio (HR) was reported as 0.28, indicating a strong protective effect. Specifically, in patients with stage IB1 cervical cancer, preoperative conization improved disease-free survival by 75%, with an HR of 0.25. For patients who underwent MIS, preoperative conization also led to a significant improvement in disease-free survival compared to those who did not have conization, with an HR of 0.21. However, it was noted that in patients who had preoperative conization, MIS increased the risk of recurrence by 34% compared to open abdominal RH, although this difference was not statistically significant (HR, 1.34; 95% CI, 0.41–4.38). The analysis found no statistical evidence that preoperative conization improved overall survival in early-stage cervical cancer patients. These results highlight the potential benefits of preoperative conization in improving disease-free survival, particularly in specific patient groups, while also indicating the need for further investigation into its effects on OS and recurrence rates. In another recently published systematic review with pairwise and network meta-analysis addressing the role of preoperative conization in early-stage cervical cancer, including 15 retrospective studies [4], the pooled results of the ten studies that compared conization and no conization showed that conization significantly reduced cancer relapse risk (HR 0.48). In network meta-analysis, treatment strategies were ranked from worst to best based on survival outcomes. MIS without preoperative conization appeared to be the worst surgical strategy, while open radical surgery with preoperative conization was the best strategy. There was no significant difference in survival outcomes among the open with conization, MIS with conization, and open without conization strategies.

Our results align with prior observational reports suggesting a survival advantage from preoperative conization in early-stage disease. A plausible explanation for observing a significant association between conization and improved OS without a corresponding statistically significant association between conization and PFS is insufficient statistical power to detect differences in PFS, potentially due to the lower-than-expected recurrence rates or loss to follow-up. Although we utilized rigorous propensity-score matching to minimize confounding, missing recurrence data or incomplete clinical assessments during follow-up may have reduced the sensitivity to detect subtle differences in progression. Consequently, an association between conization and improved PFS might exist but remain undetectable within the current study’s statistical framework. Future prospective studies with more comprehensive and prolonged follow-up may help clarify this issue.

It has been suggested that performing conization may pose challenges to RH procedures due to cervical induration and inflammation, which commonly lead surgeons to avoid the procedure. In our matched cohort study, there were no statistically significant differences in median operative time, estimated blood loss, or operative complication rates between the groups (see Table 1). These findings are consistent with existing literature, which indicates that prior conization does not increase surgical difficulty or perioperative morbidity [21,29,30,31]. This evidence suggests that, rather than complicating the surgical procedure, conization may function as a strategic intervention that potentially improves oncological outcomes.

We also observed that positive cone margins strongly predicted residual disease at hysterectomy and increased need for adjuvant therapy but did not translate into significant differences in OS or PFS within the conization group. This suggests that while a positive cone margin could reflect the extent and severity of cervical cancer, its actual impact on survival outcomes would depend on other factors such as residual cancer in the postcone uterus, surgical radicality, locoregional spread of cancer to pelvic lymph nodes and parametrium, and adjuvant treatment.

Strengths of our study include a large single-center cohort with rigorous propensity-score matching on a comprehensive set of prognostic covariates, including pelvic node, parametrial, and margin status, as well as detailed margin-residual analyses. By conducting both full-cohort and low-risk subgroup analyses, we demonstrate that the association between conization and improved OS is robust across risk strata.

Limitations warrant consideration. Given the retrospective nature of this study, it is essential to acknowledge the potential for selection bias associated with the decision to perform preoperative conization. Although we used rigorous propensity-score matching to balance measurable clinicopathologic factors, clinical judgment guiding conization, such as suspicion of microinvasion, availability of prior histology, or the desire to minimize intraoperative tumor handling, may reflect unmeasured prognostic variables that could impact outcomes. Additionally, other unmeasured confounders (e.g., HPV genotype, immune status, surgical approach, and adjuvant therapy) may still influence results despite the matching. In addition, excluding variables with more than 20% missing data (tumor grade, LVSI, depth) could introduce bias. Finally, our single-institution experience may limit generalizability, particularly in settings with different surgical or adjuvant protocols.

Future directions include prospective, multi-center studies or randomized trials to confirm causality and quantify the magnitude of the OS and PFS benefits of preoperative conization. Correlative studies incorporating molecular and immunologic markers may also elucidate the biological basis for selecting the most suitable candidates for the procedure.

## 5. Conclusions

Our propensity-score-matched analysis suggests that preoperative conization before RH is associated with improved overall survival in stage IB1 cervical cancer without a clear impact on progression-free survival. These data support the integration of conization into personalized surgical planning, with further prospective validation needed.

## Figures and Tables

**Table 1:** Baseline characteristics before and after matching in the entire study cohort.

Variable	Before Matching			After Matching		
	Conization (*n* = 314)	Control (*n* = 528)	*p*-Value	Conization (*n* = 274)	Control (*n* = 274)	*p*-Value
Age: median (IQR), years	48.5 (13.0)	47.0 (13.0)	0.157	49.0 (13.0)	47.0 (12.0)	0.763
Parity: median (IQR)	2 (1)	2 (1)	0.871	2 (1)	2 (1)	0.510
Menopause	122 (38.9%)	185 (35.0%)	0.299	107 (39.1%)	96 (35.0%)	0.376
Radical hysterectomy			0.079			0.102
Type B	18 (5.8%)	16 (3.0%)		17 (6.2%)	8 (2.9%)	
Type C	295 (94.2%)	512 (97.0%)		257 (93.8%)	266 (97.1%)	
Pathologic maximum tumor diameter: median (IQR), cm	1.6 (1.6)	2.5 (1.4)	<0.001 *	1.7 (1.7)	2.0 (1.4)	0.159
Histological type			0.016 *			0.935
Squamous	231 (73.6%)	340 (64.4%)		194 (70.8%)	195 (71.2%)	
Adeno	66 (21.0%)	140 (26.5%)		63 (23.0%)	64 (23.4%)	
Adenosquamous	17 (5.4%)	48 (9.1%)		17 (6.2%)	15 (5.5%)	
Histological grade			0.001 *			0.001 *
Well differentiated	49 (25.3%)	132 (26.9%)		47 (25.7%)	72 (28.3%)	
Moderately differentiated	130 (67.0%)	267 (54.5%)		123 (67.2%)	135 (67.2%)	
Poorly differentiated	15 (7.7%)	91 (18.6%)		13 (7.1%)	47 (7.1%)	
LVSI	171 (68.7%)	323 (72.3%)	0.362	151 (68.3%)	139 (63.2%)	0.299
Depth of stromal invasion			0.111			0.502
Inner1/3	37 (18.5%)	73 (14.3%)		35 (18.6%)	59 (22.8%)	
Middle1/3	55 (27.5%)	119 (23.3%)		49 (26.1%)	69 (26.6%)	
Outer1/3	108 (54.0%)	319 (62.4%)		104 (55.3%)	131 (50.6%)	
Vaginal metastasis	22 (7.0%)	89 (16.9%)	<0.001 *	22 (8.0%)	26 (9.5%)	0.826
Adnexal metastasis	0 (0.0%)	9 (1.7%)	0.058	0 (0.0%)	0 (0.0%)	0.662
Uterine corpus metastasis	6 (1.9%)	14 (2.7%)	0.216	6 (2.2%)	3 (1.1%)	0.383
Pelvic node metastasis	36 (11.5%)	97 (18.4%)	0.011 *	35 (12.8%)	31 (11.3%)	0.694
Parametrial metastasis	35 (11.2%)	104 (19.7%)	0.002 *	35 (12.8%)	34 (12.4%)	1.000
Positive vaginal margin	8 (2.5%)	25 (4.7%)	0.249	8 (2.9%)	7 (2.6%)	0.676
Positive parametrial margin	0 (0.0%)	5 (0.9%)	0.164	0 (0.0%)	0 (0.0%)	1.000
Operative time:median (IQR), min	210.0 (51.3)	220.0 (55.0)	0.022 *	211.0 (53.0)	220.0 (56.5)	0.273
Estimated blood loss: median (IQR), mL	400.0 (400.0)	400.0 (500.0)	0.568	400.0 (425.0)	400.0 (500.0)	0.804
Operative complications	30 (9.6%)	57 (10.8%)	0.643	28 (10.2%)	30 (10.9%)	0.890
Adjuvant treatment	98 (31.2%)	262 (50.0%)	<0.001 *	96 (35.0%)	98 (35.8%)	0.929
Adjuvant radiation			<0.001 *			0.903
Brachytherapy alone	10 (3.2%)	20 (3.8%)		10 (3.6%)	12 (4.4%)	
WPRT ± brachytherapy	83 (26.4%)	226 (43.5%)		81 (29.6%)	81 (29.7%)	
Adjuvant chemotherapy	70 (22.3%)	186 (35.5%)	<0.001 *	68 (24.8%)	63 (23.0%)	0.689
Adjuvant CCRT	9 (2.9%)	33 (6.3%)	0.039 *	9 (3.3%)	6 (2.2%)	0.606

* Statistically significant *p* < 0.05. CCRT: concurrent chemoradiation, IQR: interquartile range, LVSI: lymph-vascular space invasion, WPRT: whole pelvic radiation.

**Table 2:** Comparison of oncological outcomes between patients with and without preoperative conization.

Outcomes	Before Matching			After Matching		
	Conization (*n* = 314)	Control (*n* = 528)	*p*-Value	Conization (*n* = 274)	Control (*n* = 274)	*p*-Value
Recurrence	16 (5.1%)	54 (10.3%)	0.013 *	15 (5.5%)	23 (8.4%)	0.235
Site of recurrence			0.037 *			0.533
Pelvis	10 (3.2%)	26 (4.9%)		9 (3.3%)	14 (5.1%)	
Distant metastasis	4 (1.3%)	22 (4.2%)		4 (1.5%)	7 (2.6%)	
Pelvis + Distant metastasis	2 (0.6%)	7 (1.3%)		2 (0.7%)	3 (1.1%)	
Death	36 (11.5%)	108 (20.5%)	0.001 *	30 (10.9%)	47 (17.2%)	0.049 *
Follow time: median (IQR), months	74.7 (80.1)	73.7 (86.3)	0.384	73.6 (79.0)	73.2 (87.7)	0.834

* Statistically significant *p* < 0.05.

**Table 3:** Oncological outcomes in patients without high-risk histopathological factors, comparing those with and without preoperative conization.

Outcomes	Before Matching			After Matching		
	Conization (*n* = 238)	Control (*n* = 346)	*p*-Value	Conization (*n* = 202)	Control (*n* = 202)	*p*-Value
Recurrence	7 (3.0%)	31 (9.0%)	0.006 *	7 (3.5%)	11 (5.5%)	0.463
Site of recurrence			0.012 *			0.187
Pelvis	6 (2.5%)	18 (5.2%)		6 (3.0%)	6 (3.0%)	
Distant metastasis	1 (0.4%)	11 (3.2%)		1 (0.5%)	6 (3.0%)	
Pelvis + Distant metastasis	0 (0.0%)	3 (0.9%)		0 (0.0%)	0 (0.0%)	
Death	21 (8.8%)	62 (17.9%)	0.003 *	16 (7.9%)	29 (14.4%)	0.058
Follow time:median (IQR), months	75.8 (83.5)	74.2 (86.8)	0.179	80.9 (94.8)	74.9 (83.8)	0.209

* Statistically significant *p* < 0.05.

## Data Availability

The data presented in this study are available on request from the corresponding author (The data are not publicly available due to privacy or ethical restrictions).
